# Internet Addiction and Problem Gambling Among Japanese University Students: Comorbidity and Lifestyle Correlates

**DOI:** 10.3390/bs16050728

**Published:** 2026-05-08

**Authors:** Saimi Yamamoto, Tsukasa Yamamoto, Harumi Ejiri, Yoko Iio, Yukihiro Mori, Manato Seguchi, Yuka Aoyama, Morihiro Ito

**Affiliations:** 1 Graduate School of Life and Health Sciences, Chubu University, 1200 Matsumoto-cho, Kasugai-shi 487-8501, Aichi, Japan; 2 Department of Early Childhood Education, College of Contemporary Education, Chubu University, 1200 Matsumoto-cho, Kasugai-shi 487-8501, Aichi, Japan; 3 Center for Nursing Practicum Support, Chubu University, 1200 Matsumoto-cho, Kasugai 487-8501, Aichi, Japan; 4 Department of Nursing, College of Life and Health Sciences, Chubu University, 1200 Matsumoto-cho, Kasugai-shi 487-8501, Aichi, Japan; 5 Department of Lifelong Sports and Health Sciences, College of Life and Health Sciences, Chubu University, 1200 Matsumoto-cho, Kasugai-shi 487-8501, Aichi, Japan; 6 Support for Pioneering Research Initiated by the Next Generation (SPRING), Chubu University, 1200 Matsumoto-cho, Kasugai-shi 487-8501, Aichi, Japan; 7 Department of Clinical Engineering, College of Life and Health Sciences, Chubu University, 1200 Matsumoto-cho, Kasugai-shi 487-8501, Aichi, Japan; 8 Department of Biomedical Sciences, College of Life and Health Sciences, Chubu University, 1200 Matsumoto-cho, Kasugai-shi 487-8501, Aichi, Japan

**Keywords:** Internet addiction, problem gambling, behavioral addiction, university students, lifestyle factors, sleep, physical activity

## Abstract

Internet addiction and problem gambling represent significant public health issues among young people. A link between the two has been noted, but few studies have comprehensively examined their comorbidity, including lifestyle factors, among Japanese university students. This study clarified the prevalence of co-occurrence of Internet addiction and problem gambling among such students, identified associated factors, and examined the relationship between these conditions and lifestyle habits. A sample of 5083 Japanese university students completed the Internet Addiction Test (IAT) and South Oaks Gambling Screen (SOGS). Logistic regression was used to examine associations with demographic characteristics and lifestyle habits (sleep, physical activity, and dietary habits). Internet addiction was significantly associated with problem gambling, and the risk of problem gambling increased with IAT scores. Internet addiction was associated with being male, being in lower academic years, poor sleep quality, insufficient physical activity, and unhealthy eating. Problem gambling was associated with IAT scores, male gender, and family history of gambling. The association between lifestyle habits and problem gambling was minor. These results suggest that Internet addiction and problem gambling are not independent issues but may share a common psychological and neural basis. These findings suggest the need for a comprehensive approach in prevention and intervention.

## 1. Introduction

In recent years, behavioral addictions have gained increasing recognition as a significant public health concern, particularly among young adults (Tran et al., 2024). Among these, problem gambling and Internet-related addictive behaviors are widely reported and are considered clinically relevant conditions (American Psychiatric Association, 2022; Mihara & Higuchi, 2017). Although each has been studied independently, growing evidence suggests that they frequently co-occur and may share common psychological and neurobiological mechanisms (National Hospital Organization Kurihama Medical and Addiction Center, 2021, 2024). Despite this, few studies have comprehensively examined their comorbidity alongside lifestyle factors, particularly in non-Western populations.

Problem gambling is formally classified as a behavioral addiction in the Diagnostic and Statistical Manual of Mental Disorders, Fifth Edition, Text Revision (DSM-5-TR), distinct from substance-related disorders (American Psychiatric Association, 2022). According to a recent large-scale meta-analysis, the prevalence of problem gambling among adults is estimated at approximately 1.0–2.0% (Tran et al., 2024). Among university students, however, prevalence rates tend to be higher, with some studies reporting figures of approximately 5% or above (Mihara & Higuchi, 2017; Yokomitsu et al., 2019), suggesting that this population warrants particular attention in prevention and early intervention efforts. University students face a unique confluence of risk factors, including increased autonomy, reduced parental oversight, financial independence, and high exposure to digital environments, all of which may facilitate the onset and maintenance of addictive behaviors (Arnett, 2000).

Internet addiction, also referred to as problematic Internet use or Internet use disorder, has similarly emerged as a major health concern among young people. With the widespread adoption of smartphones and mobile technologies, daily Internet exposure has increased dramatically, and university students are among the heaviest users (Ministry of Internal Affairs and Communications Japan, 2023). Systematic reviews report a wide range of prevalence rates for Internet gaming disorder and broader Internet addiction, from 0.7% to 27.5%, with consistently higher rates observed among young men (Mihara & Higuchi, 2017).

A growing body of literature suggests that Internet addiction and problem gambling are not independent phenomena but are positively associated and potentially share a common etiological basis (National Hospital Organization Kurihama Medical and Addiction Center, 2021, 2024; Yau et al., 2014). Yau and colleagues reported that problematic Internet use was significantly associated with problem gambling severity in a large high-school sample, with each condition independently predicting the other (Yau et al., 2014). Similarly, Pallanti and colleagues demonstrated that co-occurring Internet addiction and gambling problems were associated with greater symptom severity and functional impairment than either condition alone (Pallanti et al., 2006). Furthermore, the expansion of online gambling platforms has been proposed as a structural factor that strengthens the link between Internet use and gambling behavior, as Internet access facilitates continuous and largely unregulated exposure to gambling stimuli (Critselis et al., 2013). The theoretical basis for this association has been articulated through several frameworks. Neurobiological research has implicated the mesolimbic dopamine system in both gambling disorder and Internet addiction, suggesting that shared reward dysregulation underlies both conditions (Grant et al., 2010; Potenza, 2008). Specifically, both disorders are associated with altered dopaminergic signaling in reward-related brain regions, heightened reward sensitivity, and deficits in inhibitory control (Potenza, 2008; Volkow et al., 2011).

At a psychological level, the Interaction of Person–Affect–Cognition–Execution (I-PACE) model provides a comprehensive framework for understanding how individual characteristics, affective states, cognitive biases, and failures of executive control interact to initiate and maintain addictive behaviors (Brand et al., 2019; Brand et al., 2016). This model has been applied to both Internet-use disorders and gambling disorder, and the present study is grounded in this integrative framework. In particular, during late adolescence and emerging adulthood, prefrontal cortex function is still maturing and impulse control abilities are not fully developed (Casey & Jones, 2010; Casey et al., 2008), suggesting that university students may be especially vulnerable to the development of comorbid behavioral addictions.

In addition to these psychological and neurobiological factors, lifestyle habits have been proposed as important correlates of behavioral addictions. Sleep deprivation has been linked to impaired prefrontal cortex functioning, reduced impulse control, and increased risk-seeking behavior, all of which may lower the threshold for addictive behaviors (Killgore, 2010). Similarly, insufficient physical activity has been associated with dysregulation of the dopaminergic reward system and increased vulnerability to stress-driven addictive behaviors (Brand et al., 2016). Irregular dietary habits, including meal skipping and poor nutritional quality, have also been associated with impulsivity and emotional dysregulation (Brand et al., 2016; Chaput et al., 2014). However, prior studies have rarely examined lifestyle factors in the context of comorbid Internet addiction and problem gambling simultaneously, leaving a significant gap in the literature.

Japan presents a particularly relevant and distinctive context for investigating the co-occurrence of Internet addiction and problem gambling. Despite legal restrictions on most forms of gambling, Japan hosts a wide range of quasi-legal and legally sanctioned gambling opportunities, including pachinko, public racing (horse, bicycle, boat, and motorcycle), and government-operated lotteries (National Hospital Organization Kurihama Medical and Addiction Center, 2021, 2024). Pachinko alone is estimated to generate annual revenues exceeding 14 trillion yen, and its cultural embeddedness and accessibility differ substantially from gambling contexts in Western nations (Japan Productivity Center, 2023). In addition, the proliferation of social games with gacha (randomized reward) mechanics and in-app purchases has created new contexts in which Internet use and financial risk-taking behaviors may intersect, particularly among young adults (Zendle & Cairns, 2018). These features of the Japanese gambling landscape suggest that the association between Internet addiction and problem gambling may be amplified by structural and cultural factors unique to Japan, and that findings from Western populations may not be directly generalizable to this context.

Furthermore, Japan reports among the highest rates of smartphone ownership and Internet use among developed nations, with university-aged individuals constituting one of the heaviest-use demographics (Ministry of Internal Affairs and Communications Japan, 2023). The combination of pervasive Internet access and a culturally distinctive gambling environment creates conditions in which the co-occurrence of Internet addiction and problem gambling may be particularly pronounced. To date, however, few large-scale studies have examined this comorbidity among Japanese university students, and even fewer have done so while simultaneously accounting for lifestyle factors.

The present study addresses these gaps by examining the prevalence and correlates of Internet addiction and problem gambling, and their co-occurrence, in a large sample of Japanese university students (n = 5083). The novelty of this study lies in three key contributions. First, it provides large-scale empirical evidence on the comorbidity of Internet addiction and problem gambling in a non-Western university population, extending prior research conducted predominantly in Western or East Asian gaming-focused samples. Second, it comprehensively examines a broad range of lifestyle factors—including sleep quality, physical activity, and dietary habits—alongside demographic variables, enabling a more holistic understanding of risk profiles. Third, it explicitly includes potential risk groups (subclinical problem gamblers; SOGS scores 1–4) that have been insufficiently addressed in existing research, allowing for a more nuanced, staged analysis of gambling-related risk. These contributions have direct implications for the design of integrated prevention and intervention programs targeting behavioral addictions in university settings.

## 2. Materials and Methods

### 2.1. Study Design and Participants

This was a cross-sectional study targeting undergraduate students at a large university located in Aichi Prefecture, Japan, which comprises diverse faculties. The survey was conducted from March to April 2025. The university has eight faculties, including science, humanities, and medicine, and we invited 11,230 enrolled undergraduate students to participate in the questionnaire survey during their annual health checkup at the beginning of the academic year.

In this study, 11,230 students were enrolled at the university, of whom 10,156 underwent the health checkup. Among those who attended the health checkup, 5127 students consented to participate and completed the survey, corresponding to a response rate of 50.5%. After excluding cases with missing data, 5083 participants were included in the final analysis. Missing values were addressed using complete-case analysis; no obvious systematic bias in the distribution of missing values was observed.

Although the sample size for this study was not determined based on prior hypothesis testing but rather on a census survey of all eligible participants, the study’s scale is likely sufficient to ensure stable estimates and adequate statistical power of logistic regression analysis.

This study was conducted with the approval of the Chubu University Ethics Review Committee (Approval No.: #20240102). Prior to the survey, participants were informed of the study’s purpose, the voluntary nature of participation, the absence of any disadvantage for non-participation, the assurance of anonymity, and the handling of data; submission of the questionnaire was deemed to constitute consent. This study was conducted in accordance with the Declaration of Helsinki.

### 2.2. Survey Method

Google Forms (https://www.google.com/forms/about/, last accessed 22 April 2025) was used to collect anonymous responses to an online questionnaire.

### 2.3. Survey Items

#### 2.3.1. Demographic Characteristics and Lifestyle Habits

Basic attributes surveyed included gender, academic year, faculty of enrollment, living arrangements, and membership in club activities or circles.

Regarding lifestyle habits, we assessed satisfaction with dietary habits, whether meals were nutritionally balanced, the substitution of meals with snacks, frequency of breakfast consumption, sleep quality, exercise habits, subjective health perception, and satisfaction with university life.

In addition, we investigated whether participants had any gambling experience, and for those who had, we additionally assessed whether their parents had gambling experience.

#### 2.3.2. Internet Addiction

To assess Internet addiction, we used the Internet Addiction Test (IAT) developed by Young (Young, 1998). This scale consists of 20 items, each of which is rated on a 5-point scale (1–5). The total score ranges from 20 to 100 points, with scores of 20–39 classified as the low-risk group, 40–69 as the moderate-risk group, and 70–100 as the high-risk group.

The IAT is a widely used scale with established reliability and validity (Jelenchick et al., 2012; Lai et al., 2013; Martin Herrero et al., 2024). In this study, Cronbach’s alpha was 0.923, indicating good internal consistency.

#### 2.3.3. Problem Gambling

To assess problem gambling, we used the Japanese version of the South Oaks Gambling Screen (SOGS) (Yokomitsu et al., 2015; Lesieur & Blume, 1987). This scale consists of 20 items, with a total score ranging from 0 to 20. Scores of 0 were classified as the non-problem group, 1–4 as the subclinical group, and 5 or higher as the problem group.

The SOGS is widely used for screening pathological gambling and has been confirmed to be reliable (Potenza, 2008; Yau & Potenza, 2015). Cronbach’s alpha coefficient in this study was 0.913, indicating sufficient internal consistency.

The cutoff values for the IAT and SOGS were set based on criteria whose validity has been confirmed in previous studies.

### 2.4. Statistical Analysis

First, descriptive statistics were calculated for each variable. Next, chi-square tests were conducted to examine the associations between the IAT and SOGS categories and basic demographic characteristics and lifestyle habits. Fisher’s exact test was used when cells contained expected frequencies of less than 5.

To examine the factors associated with Internet addiction and problem gambling, we performed multinomial logistic regression analysis. Variables included in the multivariate analysis were selected based on theoretical validity derived from previous studies and those with *p* < 0.10 in univariate analysis.

Regarding the IAT, because the number of cells was small in some categories, it was treated as a continuous variable in the logistic regression analysis. Odds ratios (ORs) and 95% confidence intervals (95% CIs) were calculated for each explanatory variable.

In the analysis, basic attributes such as gender and year of study were considered as confounding factors. Additionally, the variance inflation factor (VIF) was calculated to assess the impact of multicollinearity. No problematic collinearity was observed for any of the variables.

The level of statistical significance was set at a two-sided *p* < 0.05. SPSS version 31 (IBM Corp., Armonk, NY, USA) was used for statistical analysis. This study is described in accordance with the STROBE statement guidelines for reporting cross-sectional studies. Because of small cell sizes in some categories, IAT was treated as a continuous variable to ensure model stability.

To examine the robustness of findings derived from the primary categorical analyses, three sensitivity analyses were conducted. First, ordinary least squares (OLS) regression was performed with IAT total score as a continuous dependent variable to verify the direction and significance of predictors identified in the primary multinomial logistic regression. Second, binary logistic regression was performed with SOGS dichotomized as 0 (no gambling problem) versus ≥1 (any gambling problem) to examine predictors of gambling risk without relying on the three-category classification. Third, binary logistic regression was performed with SOGS dichotomized as 0–4 (non-problem/subclinical) versus ≥5 (problem gambling) to assess predictors of clinically significant gambling problems while avoiding the instability associated with the small problem gambling group (n = 14) in the primary analysis. Results of these sensitivity analyses are presented in Supplementary Tables S1–S3.

Chi-square tests were conducted as preliminary univariate screening analyses to identify candidate variables for inclusion in the subsequent logistic regression models; the two analytical approaches serve complementary rather than redundant roles, with chi-square tests informing variable selection and logistic regression providing adjusted estimates of independent associations.

## 3. Results

### 3.1. Participant Characteristics and Lifestyle Habits

Of the 5083 participants, 3484 (68.5%) were male and 1599 (31.5%) were female. By year of study, first-year students were the most numerous at 2342 (46.1%). By academic division, 2274 (44.7%) were in the sciences, 1849 (36.4%) in the humanities, and 960 (18.9%) in medical studies. Regarding living arrangements, 4159 (81.8%) lived with family or relatives, accounting for the majority.

Regarding lifestyle habits, 4781 students (94.1%) reported being satisfied with their diet; however, 1732 students (34.1%) stated they did not pay attention to nutritional balance, and 997 students (19.6%) admitted to occasionally substituting meals with snacks. With respect to breakfast frequency, 2579 individuals (50.7%) reported eating breakfast “every day,” while 376 (7.4%) reported “never” eating breakfast. In terms of sleep quality, 3497 individuals (68.8%) reported being satisfied. In addition, 2547 participants (50.1%) exercised once or twice a month or not at all. A total of 856 participants (16.8%) had experience with gambling (Table 1).

### 3.2. Association Between the Three IAT Groups and Demographic Characteristics and Lifestyle Habits

Based on IAT scores, the participants were classified into three groups: low-risk (20–39 points), medium-risk (40–69 points), and high-risk (70–100 points). The low-risk group consisted of 2607 participants, the medium-risk group of 2312, and the high-risk group of 164.

Analysis of the associations between the three IAT groups and various variables revealed significant correlations with year of study (*p* = 0.011), affiliation (*p* < 0.001), participation in club activities or circles (*p* < 0.001), satisfaction with dietary habits (*p* < 0.001), consumption of nutritionally balanced meals (*p* < 0.001), the habit of substituting meals with snacks (*p* < 0.001), frequency of breakfast consumption (*p* < 0.001), sleep quality (*p* < 0.001), frequency of exercise (*p* < 0.001), health status over the past month (*p* < 0.001), and satisfaction with university life (*p* < 0.001; Table 2). In contrast, no significant associations were found with gender, living arrangements, or health checkup status.

In comparisons between groups, the IAT high-risk group had a higher proportion of individuals who were not conscious of nutritional balance, who substituted meals with snacks, who skipped breakfast, who were dissatisfied with their sleep quality, and who had low exercise frequency. Furthermore, the IAT high-risk group also had a higher proportion of individuals who reported poor health status over the past month and who were dissatisfied with their university life.

### 3.3. Association Between the Three SOGS Groups and Basic Demographics, Lifestyle Habits, and Internet Addiction

When the 856 participants with gambling experience were classified based on their SOGS scores, 380 (44.4%) were in the non-problem group (0 points), 462 (54.0%) were in the potential problem group (1–4 points), and 14 (1.6%) were in the problem group (5–17 points).

Analysis of the associations between the three SOGS groups and various variables revealed significant correlations with the three IAT groups (*p* < 0.001), gender (*p* < 0.001), year of study (*p* = 0.022), satisfaction with dietary habits (*p* = 0.021), eating nutritionally balanced meals (*p* = 0.001), the habit of substituting meals with snacks (*p* = 0.015), and the presence of parental gambling problems (*p* < 0.001; Table 3). In contrast, no significant associations were found for affiliation, living arrangements, participation in club activities or circles, frequency of breakfast, sleep quality, frequency of exercise, health status over the past month, satisfaction with university life, or health checkup status.

Regarding the association with the IAT, the proportion of the moderate-risk group was higher in the SOGS problem group, indicating a link between Internet addiction tendencies and problem gambling. Furthermore, all members of the problem group were male, and the proportion of upperclassmen was high. Additionally, the proportion of students with parental gambling problems tended to be higher in the SOGS problem group.

### 3.4. Logistic Regression Analysis

#### 3.4.1. Factors Associated with Internet Addiction

The results of the multinomial logistic regression analysis with the IAT as the dependent variable are shown in Table 4. Males had significantly higher odds of belonging to the moderate-risk group (OR = 1.199, 95% CI: 1.044–1.376, *p* = 0.010) and the high-risk group (OR = 1.663, 95% CI: 1.116–2.478, *p* = 0.012) compared to females.

By year of study, first-, second-, and third-year students all had significantly higher odds of belonging to the moderate-risk group compared to fourth-year students (first-year: OR = 1.563, 95% CI: 1.301–1.878, *p* < 0.001; second-year: OR = 1.284, 95% CI: 1.050–1.569, *p* = 0.015; third-year students: OR = 1.246, 95% CI: 1.005–1.546, *p* = 0.045). Conversely, no significant association with year of study was observed in the high-risk group.

Regarding lifestyle habits, eating nutritionally balanced meals was associated with reduced odds in the moderate-risk group (OR = 0.754, 95% CI: 0.662–0.859, *p* < 0.001) and the high-risk group (OR = 0.505, 95% CI: 0.354–0.720, *p* < 0.001). Substituting meals with snacks was associated with an increased odds ratio in the moderate-risk group (OR = 1.924, 95% CI: 1.645–2.250, *p* < 0.001) and the high-risk group (OR = 3.786, 95% CI: 2.644–5.422, *p* < 0.001). Satisfaction with sleep quality was associated with a reduced odds ratio in the moderate-risk group (OR = 0.668, 95% CI: 0.586–0.761, *p* < 0.001) and the high-risk group (OR = 0.575, 95% CI: 0.406–0.815, *p* = 0.002). Furthermore, exercising once or twice a month or not at all was associated with an increased odds ratio in the moderate-risk group (OR = 1.266, 95% CI: 1.117–1.435, *p* < 0.001) and the high-risk group (OR = 2.049, 95% CI: 1.406–2.986, *p* < 0.001).

Poor health status in the past month was associated with increased odds in the moderate-risk group (OR = 1.687, 95% CI: 1.366–2.084, *p* < 0.001) and the high-risk group (OR = 2.744, 95% CI: 1.798–4.188, *p* < 0.001), while satisfaction with college life was associated with decreased odds in the moderate-risk group (OR = 0.573, 95% CI: 0.457–0.718, *p* < 0.001) and the high-risk group (OR = 0.309, 95% CI: 0.201–0.476, *p* < 0.001).

#### 3.4.2. Factors Associated with Problem Gambling

The results of the multinomial logistic regression analysis with SOGS as the dependent variable are shown in Table 5. IAT scores were significantly associated with both the latent problem group (OR = 1.015, 95% CI: 1.005–1.025, *p* = 0.004) and the problem group (OR = 1.060, 95% CI: 1.019–1.102, *p* = 0.003). That is, the higher the IAT score, the higher the risk of problem gambling.

In terms of year of study, second-year students had significantly higher odds of belonging to the potential problem group compared to fourth-year students (OR = 1.659, 95% CI: 1.110–2.480, *p* = 0.014). In contrast, in the problem group, the odds for second-year students were significantly lower (OR = 0.103, 95% CI: 0.011–0.958, *p* = 0.046).

Furthermore, satisfaction with one’s diet was associated with lower odds of belonging to the problem group (OR = 0.147, 95% CI: 0.023–0.948, *p* = 0.044). In contrast, eating a nutritionally balanced diet was associated with increased odds of belonging to the problem gambling group (OR = 11.611, 95% CI: 1.321–102.037, *p* = 0.027). However, this estimate had a wide confidence interval and was highly uncertain. Furthermore, the presence of parental gambling problems was also significantly associated with problem gambling.

## 4. Discussion

This study comprehensively examined the comorbidity of Internet addiction and problem gambling, as well as associated factors, among Japanese university students. The results indicated that a tendency toward Internet addiction was significantly associated with problem gambling, and that a higher tendency toward Internet addiction was associated with a higher risk of problem gambling. Furthermore, it was revealed that lifestyle habits such as sleep quality and physical activity were associated with Internet addiction. These results support the possibility that both are interrelated behavioral addictions. Importantly, sensitivity analyses using continuous and alternative binary operationalizations of both IAT and SOGS yielded results consistent with the primary categorical analyses (Supplementary Tables S1–S3), supporting the robustness of the findings.

First, regarding the association between Internet addiction and problem gambling, the findings of this study are consistent with previous research. The association between problematic Internet use and problem gambling has already been reported (Yau et al., 2014), and it has been shown that gamblers with co-occurring Internet addiction exhibit more pronounced symptom severity and psychological problems (Pallanti et al., 2006). Furthermore, it has been suggested that the development of the Internet environment has made access to gambling activities easier, potentially strengthening the association between the two (Critselis et al., 2013). This study illustrated a similar association in the specific population of Japanese university students, thereby supporting and expanding existing knowledge.

Such associations can be explained by a common psychological and neurobiological basis. Behavioral addictions have been linked to the reward system, particularly the dopamine system, suggesting that gambling disorder and Internet addiction may share a common neural basis (Grant et al., 2010). Furthermore, the I-PACE model posits that addictive behaviors are formed and maintained through the interaction of personal characteristics, emotions, cognition, and executive functions (Brand et al., 2019). The results of this study are consistent with these theoretical frameworks and suggest the need to understand Internet addiction and problem gambling as behavioral addictions based on a common decline in self-control functions and increased reward sensitivity.

In particular, it is known that at college age, the prefrontal cortex function is still maturing, and impulse control abilities are not fully developed (Casey & Jones, 2010; Casey et al., 2008). In addition, high exposure to smartphones and online environments may facilitate access to rewarding stimuli and promote the co-occurrence of behavioral addictions. Therefore, the associations demonstrated in this study are likely the result of the interaction between individual and environmental factors.

Regarding associations with lifestyle habits, this study found that poor sleep quality and insufficient physical activity were significantly associated with Internet addiction. These results are consistent with previous studies suggesting that sleep deprivation and lack of exercise may influence behavioral and psychological processes related to addictive behaviors (Brand et al., 2016; Chaput et al., 2014). Sleep deprivation is known to be associated with impaired prefrontal cortex function, which may increase impulsivity and impair self-control. Furthermore, physical activity is involved in stress regulation and the regulation of the reward system; a lack thereof may increase the risk of addictive behaviors. These findings suggest that improving lifestyle habits is important for the prevention of and intervention against Internet addiction.

In contrast, in this study, the association between problem gambling and lifestyle habits was limited. This suggests that gambling behavior may be more strongly influenced by psychological and social factors than by lifestyle habits. Indeed, in this study, parental gambling problems were associated with problem gambling, suggesting the involvement of family environment and genetic factors.

A particularly noteworthy finding was that being conscious of dietary nutritional balance was positively associated with problem gambling. While this result may initially appear contradictory, it can be interpreted from several perspectives. First, as this study is cross-sectional, reverse causality is a possibility. That is, students with a tendency toward problem gambling may have been more concerned about their own health, and as a result, may have responded that they were “conscious of nutritional balance.”

Second, the problem gambling group in this study was very small (n = 14), and the instability of the regression estimates is a concern. The findings derived from this group should therefore be regarded as exploratory and preliminary rather than definitive. In particular, given the wide confidence intervals observed in the primary analysis, the possibility of overestimation due to sparse data bias cannot be excluded. The sensitivity analysis using binary logistic regression with SOGS dichotomized as 0–4 versus ≥5 (Supplementary Table S3) yielded more stable estimates and similarly confirmed the significant association between IAT scores and problem gambling risk, as well as the role of parental gambling history.

Furthermore, these results can also be explained from the perspective of the “health consciousness paradox.” That is, even individuals who engage in unhealthy behaviors do not necessarily have poor health awareness; rather, they may consciously engage in healthy behaviors as a form of compensation. Similarly, college students may perceive themselves as being health-conscious regarding their diet even while exhibiting Internet addiction or gambling behaviors. Therefore, the association observed in this study may reflect compensatory health awareness rather than actual health behaviors. This interpretation remains speculative and requires further investigation.

In Japan, a unique gambling environment exists, including in-game purchases in social games and pachinko, and a potential association between Internet use and financial risk-taking behaviors has been noted. With increasing access to online gambling, these factors may be related to the association between Internet addiction and problem gambling. The present study identified such an association among Japanese university students, with findings generally consistent with previous studies conducted in Western populations. These results may suggest the involvement of environmental factors and have implications for integrated preventive approaches targeting young adults.

This study has several limitations. First, as a cross-sectional study it cannot establish causality. Second, because the sample consists of students from a single university, generalizability is limited. However, as the study was conducted at a large university encompassing multiple faculties, some diversity was present. Third, the sample size for the problem gambling group was small, resulting in uncertainty regarding some estimates. These results should be interpreted with caution due to the small sample size of the problem group. Fourth, because psychological factors (such as depression and impulsivity) were not assessed, the influence of confounding factors may not have been fully eliminated. Fifth, because self-administered questionnaires were used, the influence of reporting bias cannot be excluded. Sixth, due to the small sample size in the problem gambling group, the statistical robustness of some analytical results may be limited. To address concerns regarding the categorical treatment of IAT and SOGS scores, sensitivity analyses were conducted using continuous and alternative binary operationalizations of these measures. The direction and significance of key predictors were consistent across all analytical approaches, supporting the robustness of the primary findings (Supplementary Tables S1–S3).

Despite these limitations, this study is valuable in that it demonstrated the association between Internet addiction and problem gambling in a large sample of college students. By simultaneously examining the comorbidity of behavioral addictions and their association with lifestyle habits, this study provides new evidence that complements existing research.

Future studies are needed to clarify causal relationships through longitudinal and multi-center studies, as well as to validate comprehensive models that incorporate psychological and environmental factors. Furthermore, mechanisms underlying the link between Internet addiction and problem gambling could be elucidated in greater detail through mediation analyses and structural equation modeling.

From a practical perspective, rather than treating Internet addiction and problem gambling as separate issues, it is necessary to evaluate and intervene in them integrally as behavioral addictions sharing a common foundation. In particular, for preventive interventions targeting university students, it is important to develop comprehensive programs that combine education on digital usage and financial behaviors with improvements in lifestyle habits such as sleep and physical activity.

## Figures and Tables

**Table 1:** Summary of participant characteristics.

Variables		n = 5083	
		n	%
Gender	Male	3484	68.5
	Female	1599	31.5
Year of study	1st year	2342	46.1
	2nd year	1197	23.5
	3rd year	824	16.2
	4th year	720	14.2
Affiliation	Liberal Arts	1849	36.4
	Science	2274	44.7
	Medical	960	18.9
Living arrangement	With family or relatives	4159	81.8
	Living alone	850	16.7
	Dorm	60	1.2
	Living with friends	9	0.2
	Other	5	0.1
Membership in club activities or circles	Sports	583	11.5
	Non-sports	694	13.7
	Independent	3806	74.9
Satisfaction with diet	Yes (Satisfied)	4781	94.1
	No (Not satisfied)	302	5.9
Meals with a focus on nutritional balance	Yes (I am mindful of it)	3351	65.9
	No (Not conscious of it)	1732	34.1
Replacing meals with snacks	Yes	997	19.6
	No	4086	80.4
Breakfast frequency	Never	376	7.4
	1–2 times a week	497	9.8
	3–4 times a week	621	12.2
	5–6 times a week	1010	19.9
	Eat every day	2579	50.7
Sleep quality	(Very/Somewhat) satisfied	3497	68.8
	Not satisfied	1586	31.2
How much do you usually exercise?	1–2 times a month/Not at all	2547	50.1
	1–2 times a week/3–4 times a week	2536	49.9
Health status over the past month	Not healthy	524	10.3
	In good health	4559	89.7
Satisfaction with college life	Yes (Satisfied)	4644	91.4
	None (Not satisfied)	439	8.6
University health checkup received within the past month	Yes	3780	74.4
	No	1303	25.6
Gambling experience	Yes	856	16.8
	None	4227	83.2

**Table 2:** Relationship between attributes and Internet dependency.

			IAT			*p*-Value
		Overall	Low-Risk Group (20–39 Points)	Moderate-Risk Group (40–69 Points)	High-Risk Group (70–100 Points)	
		n = 5083	n = 2607	n = 2312	n = 164	
		n (%)	n (%)	n (%)	n (%)	
Gender	Male	3484 (68.5)	1762 (67.6)	1606 (69.5)	116 (70.7)	n.s.
	Female	1599 (31.5)	845 (32.4)	706 (30.5)	48 (29.3)	
Year of study	1st year	2342 (46.1)	1150 (44.1)	1119 (48.4)	73 (44.5)	0.011
	2nd year	1197 (23.5)	619 (23.7)	538 (23.3)	40 (24.4)	
	3rd year	824 (16.2)	424 (16.3)	371 (16.0)	29 (17.7)	
	4th year	720 (14.2)	414 (15.9)	284 (12.3)	22 (13.4)	
Affiliation	Liberal Arts	1849 (36.4)	972 (37.3)	816 (35.3)	61 (37.2)	<0.001
	Science	2274 (44.7)	1095 (42.0)	1100 (47.6)	79 (48.2)	
	Medical	960 (18.9)	540 (20.7)	396 (17.1)	24 (14.6)	
Living arrangement	With family or relatives	4159 (81.8)	2137 (82.0)	1884 (81.5)	138 (84.1)	n.s.
	Living alone	850 (16.7)	429 (16.5)	398 (17.2)	23 (14.0)	
	Dorm	60 (1.2)	33 (1.3)	24 (1.0)	3 (1.8)	
	Living with friends	9 (0.2)	6 (0.2)	3 (0.1)	0 (0)	
	Other	5 (0.1)	2 (0.1)	3 (0.1)	0 (0)	
Membership in club activities or circles	Sports	583 (11.5)	351 (13.5)	218 (9.4)	14 (8.5)	<0.001
	Non-athletic	694 (13.7)	343 (13.2)	326 (14.1)	25 (15.2)	
	Independent	3806 (74.9)	1913 (73.4)	1768 (76.5)	125 (76.2)	
Satisfaction with diet	Satisfied	4781 (94.1)	2485 (95.3)	2165 (93.6)	131 (79.9)	<0.001
	Dissatisfied	302 (5.9)	122 (4.7)	147 (6.4)	33 (20.1)	
Meals with a focus on nutritional balance	Yes (I am mindful of it)	3351 (65.9)	1883 (72.2)	1400 (60.6)	68 (41.5)	<0.001
	No (Not conscious of it)	1732 (34.1)	724 (27.8)	912 (39.4)	96 (58.5)	
Replacing meals with snacks	Yes	997 (19.6)	355 (13.6)	571 (24.7)	71 (43.3)	<0.001
	None	4086 (80.4)	2252 (86.4)	1741 (75.3)	93 (56.7)	
Frequency of breakfast	Never	376 (7.4)	169 (6.5)	186 (8.0)	21 (12.8)	<0.001
	1–2 times a week	497 (9.8)	248 (9.5)	230 (9.9)	19 (11.6)	
	3–4 times a week	621 (12.2)	302 (11.6)	296 (12.8)	23 (14.0)	
	5–6 times a week	1010 (19.9)	452 (17.3)	528 (22.8)	30 (18.3)	
	Eat every day	2579 (50.7)	1436 (55.1)	1072 (46.4)	71 (43.3)	
Sleep quality	(Very/Somewhat) satisfied	3497 (68.8)	1959 (75.1)	1455 (62.9)	83 (50.6)	<0.001
	Dissatisfied	1586 (31.2)	648 (24.9)	857 (37.1)	81 (49.4)	
How much do you usually exercise?	1–2 times a month/ Not exercising at all	2547 (50.1)	1166 (44.7)	1268 (54.8)	113 (68.9)	<0.001
	1–2 times a week/ 3–4 times a week	2536 (49.9)	1441 (55.3)	1044 (45.2)	51 (31.1)	
Health status over the past month	Not healthy	524 (10.3)	168 (6.4)	309 (13.4)	47 (28.7)	<0.001
	Healthy	4559 (89.7)	2439 (93.6)	2003 (86.6)	117 (71.3)	
Satisfaction with college life	Satisfied	4644 (91.4)	2468 (94.7)	2054 (88.8)	122 (74.4)	<0.001
	Dissatisfied	439 (8.6)	139 (5.3)	258 (11.2)	42 (25.6)	
University health checkup received within the past month	Yes	3780 (74.4)	1964 (75.3)	1694 (73.3)	122 (74.4)	n.s.
	No	1303 (25.6)	643 (24.7)	618 (26.7)	42 (25.6)	

The *p*-values were calculated using a chi-square test or Fisher’s exact tests, as appropriate. IAT, Internet Addiction Test, n.s., no significant difference.

**Table 3:** Association between the three SOGS groups and basic characteristics, lifestyle habits, and Internet addiction.

			SOGS			*p*-Value
		Overall	Non-Problem Group (0)	Potential Problem Group (1–4)	Problem Group (5–17)	
		n = 856	n = 380, 44.4%	n = 462, 54.0%	n = 14, 1.6%	
		n (%)	n (%)	n (%)	n (%)	
IAT	20–39 points	436 (50.9)	217 (57.1)	216 (46.8)	3 (21.4)	<0.001
	40–69 points	381 (44.5)	154 (40.5)	216 (46.8)	11 (78.6)	
	70–100 points	39 (4.6)	9 (2.4)	30 (6.5)	0 (0)	
Gender	Male	768 (89.7)	324 (85.3)	430 (93.1)	14 (100)	<0.001
	Female	88 (10.3)	56 (14.7)	32 (6.9)	0 (0)	
Year of study	1st year	199 (23.2)	83 (21.8)	115 (24.9)	1 (7.1)	0.022
	2nd year	238 (27.8)	97 (25.5)	140 (30.3)	1 (7.1)	
	3rd year	231 (27.0)	107 (28.2)	119 (25.8)	5 (35.7)	
	4th year	188 (22.0)	93 (24.5)	88 (19.0)	7 (50.0)	
Affiliation	Liberal Arts	301 (35.2)	145 (38.2)	152 (32.9)	4 (28.6)	n.s.
	Science	402 (47.0)	167 (43.9)	228 (49.4)	7 (50.0)	
	Medical	153 (17.9)	68 (17.9)	82 (17.7)	3 (21.4)	
Living arrangement	With family or relatives	698 (81.5)	319 (83.9)	367 (79.4)	12 (85.7)	n.s.
	Living alone	146 (17.1)	56 (14.7)	88 (19.0)	2 (14.3)	
	Dorm	8 (0.9)	3 (0.8)	5 (1.1)	0 (0)	
	Living with friends	2 (0.2)	1 (0.3)	1 (0.2)	0 (0)	
	Other	2 (0.2)	1 (0.3)	1 (0.2)	0 (0)	
Membership in club activities or circles	Sports	129 (15.1)	56 (14.7)	70 (15.2)	3 (21.4)	n.s.
	Non-athletic	115 (13.4)	58 (15.3)	54 (11.7)	3 (21.4)	
	Independent	612 (71.5)	266 (70.0)	338 (73.2)	8 (57.1)	
Satisfaction with diet	Satisfied	797 (93.1)	363 (95.5)	422 (91.3)	12 (85.7)	0.021
	Not satisfied	59 (6.9)	17 (4.5)	40 (8.7)	2 (14.3)	
Meals with a focus on nutritional balance	Yes (I am mindful of it)	544 (63.6)	259 (68.2)	272 (58.9)	13 (92.9)	0.001
	No (Not conscious of it)	312 (36.4)	121 (31.8)	190 (41.1)	1 (7.1)	
Replacing meals with snacks	Yes	163 (19.0)	58 (15.3)	104 (22.5)	1 (7.1)	0.015
	None	693 (81.0)	322 (84.7)	358 (77.5)	13 (92.9)	
Frequency of breakfast	Never	102 (11.9)	37 (9.7)	63 (13.6)	2 (14.3)	n.s.
	1–2 times a week	114 (13.3)	39 (10.3)	73 (15.8)	2 (14.3)	
	3–4 times a week	146 (17.1)	67 (17.6)	76 (16.5)	3 (21.4)	
	5–6 times a week	165 (19.3)	72 (18.9)	89 (19.3)	4 (28.6)	
	Eat every day	329 (38.4)	165 (43.4)	161 (34.8)	3 (21.4)	
Sleep quality	(Very/Somewhat) satisfied	544 (63.6)	248 (65.3)	286 (61.9)	10 (71.4)	n.s.
	Not satisfied	312 (36.4)	132 (34.7)	176 (38.1)	4 (28.6)	
How much do you usually exercise?	1–2 times a month/Not at all	392 (45.8)	177 (46.6)	211 (45.7)	4 (28.6)	n.s.
	1–2 times a week/ 3–4 times a week	464 (54.2)	203 (53.4)	251 (54.3)	10 (71.4)	
Health status over the past month	Not healthy	115 (13.4)	42 (11.1)	71 (15.4)	2 (14.3)	n.s.
	Healthy	741 (86.6)	338 (88.9)	391 (84.6)	12 (85.7)	
Satisfaction with college life	Satisfied	769 (89.8)	349 (91.8)	407 (88.1)	13 (92.9)	n.s.
	Not satisfied	87 (10.2)	31 (8.2)	55 (11.9)	1 (7.1)	
University health checkup received within the past month	Yes	691 (80.7)	306 (80.5)	377 (81.6)	8 (57.1)	n.s.
	No	165 (19.3)	74 (19.5)	85 (18.4)	6 (42.9)	
Do or did your parents have a gambling problem?	No problem	775 (90.5)	360 (94.7)	407 (88.1)	8 (57.1)	<0.001
	Either parent had or has problems	62 (7.2)	17 (4.5)	43 (9.3)	2 (14.3)	
	parents have/had problems	19 (2.2)	3 (0.8)	12 (2.6)	4 (28.6)	

The *p*-values were calculated using a chi-square test or Fisher’s exact tests, as appropriate. SOGS, South Oaks Gambling Screen; IAT, Internet Addiction Test, n.s., no significant difference.

**Table 4:** Results of the multinomial logistic regression analysis with the IAT as the dependent variable.

		IAT							
		Moderate-Risk Group (40–69 Points)				High-Risk Group (70–100 Points)			
Variable	Category	OR	OR 95% CI		*p*-Value	OR	OR 95% CI		*p*-Value
			Lower	Upper			Lower	Upper	
Gender (ref. Female)	Male	1.199	1.044	1.376	0.010	1.663	1.116	2.478	0.012
Year of study (ref. 4th year)	1st year	1.563	1.301	1.878	<0.001	1.655	0.974	2.812	0.063
	2nd year	1.284	1.050	1.569	0.015	1.296	0.735	2.284	0.370
	3rd year	1.246	1.005	1.546	0.045	1.380	0.756	2.520	0.294
Affiliation (ref. Medical)	Liberal Arts	1.048	0.886	1.240	0.586	1.132	0.680	1.886	0.633
	Science	1.182	0.997	1.400	0.054	1.180	0.706	1.973	0.527
Membership in club activities or circles (ref. no membership)	Sports-related	0.852	0.701	1.035	0.106	1.089	0.589	2.013	0.787
	Non-sports	1.059	0.889	1.261	0.522	1.205	0.752	1.932	0.439
Satisfaction with diet (ref. not satisfied)	Satisfied	1.151	0.879	1.507	0.305	0.523	0.320	0.854	0.010
Meals with a focus on nutritional balance (ref. no)	Yes	0.754	0.662	0.859	<0.001	0.505	0.354	0.720	<0.001
Replacing meals with snacks (ref. no)	Yes	1.924	1.645	2.250	<0.001	3.786	2.644	5.422	<0.001
Frequency of breakfast (ref. every day)	Never	1.075	0.846	1.366	0.556	0.917	0.518	1.623	0.767
	1–2 times a week	1.005	0.815	1.239	0.960	0.773	0.442	1.354	0.368
	3–4 times a week	1.164	0.963	1.406	0.117	0.883	0.523	1.490	0.641
	5–6 times a week	1.401	1.201	1.634	<0.001	0.995	0.631	1.569	0.982
Sleep quality (ref. not satisfied)	(Very/Somewhat) satisfied	0.668	0.586	0.761	<0.001	0.575	0.406	0.815	0.002
How much do you usually exercise (ref. 3–4 times per week)	1–2 times a month/ I don’t exercise at all	1.266	1.117	1.435	<0.001	2.049	1.406	2.986	<0.001
Health status over the past month (ref. Healthy)	Not healthy	1.687	1.366	2.084	<0.001	2.744	1.798	4.188	<0.001
Satisfaction with college life (ref. not satisfied)	Satisfied	0.573	0.457	0.718	<0.001	0.309	0.201	0.476	<0.001

Calculated using multinomial logistic regression analysis. IAT, Internet Addiction Test OR, odds ratio; CI, confidence interval.

**Table 5:** Results of multinomial logistic regression analysis with SOGS as the dependent variable.

SOGS 3-Group Classification		SOGS							
		Potential Problem Group				Problem Group			
		OR	OR 95% CI		*p*-Value	OR	OR 95% CI		*p*-Value
			Lower	Upper			Lower	Upper	
Predictors of SOGS									
IAT		1.015	1.005	1.025	0.004	1.060	1.019	1.102	0.003
Year of study (ref. 4th grade)	1st year	1.480	0.975	2.244	0.065	0.141	0.015	1.297	0.083
	2nd year	1.659	1.110	2.480	0.014	0.103	0.011	0.958	0.046
	3rd year	1.196	0.800	1.788	0.383	0.530	0.139	2.025	0.353
Satisfaction with diet (ref. not satisfied)	Satisfied	0.577	0.316	1.054	0.074	0.147	0.023	0.948	0.044
Meals with a focus on nutritional balance (ref. no)	Yes	0.761	0.565	1.025	0.072	11.611	1.321	102.037	0.027
Replacing meals with snacks (ref. no)	Yes	1.421	0.980	2.058	0.064	0.135	0.013	1.426	0.096
Does/did either of your parents have a gambling problem? (ref. parents have/had problems)	No problem	0.290	0.079	1.066	0.062	0.019	0.003	0.113	<0.001
	Either parent had or has problems	0.575	0.140	2.363	0.443	0.089	0.009	0.906	0.041

Calculated using multinomial logistic regression analysis. SOGS, South Oaks Gambling Screen; OR, odds ratio; CI, confidence interval.

## Data Availability

The data presented in this study are available on request from the corresponding author due to privacy and ethical constraints.

## Supplementary Materials

The following supporting information can be downloaded at: https://www.mdpi.com/article/10.3390/bs16050728/s1, Table S1: Linear regression analysis with IAT total score as a continuous dependent variable (sensitivity analysis); Table S2: Binary logistic regression analysis with SOGS dichotomized as 0 (no problem) vs. ≥1 (any gambling problem) as the dependent variable (sensitivity analysis); Table S3: Binary logistic regression analysis with SOGS dichotomized as 0–4 (non/subclinical) vs. ≥5 (problem gambling) as the dependent variable (sensitivity analysis).

## References

[B1-behavsci-16-00728] American Psychiatric Association (2022). Diagnostic and statistical manual of mental disorders.

[B2-behavsci-16-00728] Arnett J. J. (2000). Emerging adulthood. A theory of development from the late teens through the twenties. American Psychologist.

[B3-behavsci-16-00728] Brand M., Wegmann E., Stark R., Muller A., Wolfling K., Robbins T. W., Potenza M. N. (2019). The Interaction of Person-Affect-Cognition-Execution (I-PACE) model for addictive behaviors: Update, generalization to addictive behaviors beyond internet-use disorders, and specification of the process character of addictive behaviors. Neuroscience & Biobehavioral Reviews.

[B4-behavsci-16-00728] Brand M., Young K. S., Laier C., Wolfling K., Potenza M. N. (2016). Integrating psychological and neurobiological considerations regarding the development and maintenance of specific Internet-use disorders: An Interaction of Person-Affect-Cognition-Execution (I-PACE) model. Neuroscience & Biobehavioral Reviews.

[B5-behavsci-16-00728] Casey B. J., Jones R. M. (2010). Neurobiology of the adolescent brain and behavior: Implications for substance use disorders. Journal of the American Academy of Child & Adolescent Psychiatry.

[B6-behavsci-16-00728] Casey B. J., Jones R. M., Hare T. A. (2008). The adolescent brain. Annals of the New York Academy of Sciences.

[B7-behavsci-16-00728] Chaput J. P., Carson V., Gray C. E., Tremblay M. S. (2014). Importance of all movement behaviors in a 24 h period for overall health. International Journal of Environmental Research and Public Health.

[B8-behavsci-16-00728] Critselis E., Janikian M., Paleomilitou N., Oikonomou D., Kassinopoulos M., Kormas G., Tsitsika A. (2013). Internet gambling is a predictive factor of Internet addictive behavior. Journal of Behavioral Addictions.

[B9-behavsci-16-00728] Grant J. E., Potenza M. N., Weinstein A., Gorelick D. A. (2010). Introduction to behavioral addictions. The American Journal of Drug and Alcohol Abuse.

[B10-behavsci-16-00728] Japan Productivity Center (2023). Leisure white paper 2023.

[B11-behavsci-16-00728] Jelenchick L. A., Becker T., Moreno M. A. (2012). Assessing the psychometric properties of the Internet Addiction Test (IAT) in US college students. Psychiatry Research.

[B12-behavsci-16-00728] Killgore W. D. (2010). Effects of sleep deprivation on cognition. Progress in Brain Research.

[B13-behavsci-16-00728] Lai C. M., Mak K. K., Watanabe H., Ang R. P., Pang J. S., Ho R. C. (2013). Psychometric properties of the internet addiction test in Chinese adolescents. Journal of Pediatric Psychology.

[B14-behavsci-16-00728] Lesieur H. R., Blume S. B. (1987). The South Oaks Gambling Screen (SOGS): A new instrument for the identification of pathological gamblers. American Journal of Psychiatry.

[B15-behavsci-16-00728] Martin Herrero J. A., Fernandez Mora K., Molina Fernandez A. (2024). Assessing the reliability, dimensions, and variance of young’s internet addiction test by applying it to adolescents at the national psychiatric hospital in costa rica. Actas Espanolas de Psiquiatria.

[B16-behavsci-16-00728] Mihara S., Higuchi S. (2017). Cross-sectional and longitudinal epidemiological studies of Internet gaming disorder: A systematic review of the literature. Psychiatry and Clinical Neurosciences.

[B17-behavsci-16-00728] Ministry of Internal Affairs and Communications Japan (2023). Information and communications in Japan: White paper 2023.

[B18-behavsci-16-00728] National Hospital Organization Kurihama Medical and Addiction Center (2021). The survey on gambling disorder and gambling related problems.

[B19-behavsci-16-00728] National Hospital Organization Kurihama Medical and Addiction Center (2024). The survey on gambling disorder and gambling-related problems.

[B20-behavsci-16-00728] Pallanti S., Bernardi S., Quercioli L. (2006). The shorter PROMIS questionnaire and the internet addiction scale in the assessment of multiple addictions in a high-school population: Prevalence and related disability. CNS Spectrums.

[B21-behavsci-16-00728] Potenza M. N. (2008). The neurobiology of pathological gambling and drug addiction: An overview and new findings. Transactions of the Royal Society B: Biological Sciences.

[B22-behavsci-16-00728] Tran L. T., Wardle H., Colledge-Frisby S., Taylor S., Lynch M., Rehm J., Volberg R., Marionneau V., Saxena S., Bunn C., Farrell M., Degenhardt L. (2024). The prevalence of gambling and problematic gambling: A systematic review and meta-analysis. Lancet Public Health.

[B23-behavsci-16-00728] Volkow N. D., Wang G. J., Fowler J. S., Tomasi D., Telang F. (2011). Addiction: Beyond dopamine reward circuitry. Proceedings of the National Academy of Sciences USA.

[B24-behavsci-16-00728] Yau Y. H., Pilver C. E., Steinberg M. A., Rugle L. J., Hoff R. A., Krishnan-Sarin S., Potenza M. N. (2014). Relationships between problematic internet use and problem-gambling severity: Findings from a high-school survey. Addictive Behaviors.

[B25-behavsci-16-00728] Yau Y. H., Potenza M. N. (2015). Gambling disorder and other behavioral addictions: Recognition and treatment. Harvard Review of Psychiatry.

[B26-behavsci-16-00728] Yokomitsu K., Sakai T., Irie T., Tayama J., Furukawa H., Himachi M., Kanazawa J., Koda M., Kunisato Y., Matsuoka H., Takada T., Takahashi F., Takahashi T., Osawa K. (2019). Gambling symptoms, behaviors, and cognitive distortions in Japanese university students. Substance Abuse Treatment, Prevention, and Policy.

[B27-behavsci-16-00728] Yokomitsu K., Takahashi T., Kanazawa J., Sakano Y. (2015). Development and validation of the Japanese version of the Gambling Related Cognitions Scale (GRCS-J). Asian Journal of Gambling Issues and Public Health.

[B28-behavsci-16-00728] Young K. S. (1998). Caught in the net: How to recognize the signs of Internet addiction—And a winning strategy for recovery.

[B29-behavsci-16-00728] Zendle D., Cairns P. (2018). Video game loot boxes are linked to problem gambling: Results of a large-scale survey. PLoS ONE.

