# Supplementary material for: Internet Addiction and Problem Gambling Among Japanese University Students: Comorbidity and Lifestyle Correlates
Source: Behav Sci (Basel). 2026 May 8;16(5):728. doi: 10.3390/bs16050728 (PMC13203873; doi:10.3390/bs16050728)
Supplement: Supplementary file 1 [file behavsci-16-00728-s001.zip › Supplementary Table S1.pdf]

**Supplementary Table S1. Linear regression analysis with IAT total score as a continuous dependent variable (sensitivity analysis).**

| Variable | Category | B | SE | β | t | p-value |
|---|---|---|---|---|---|---|
| (Constant) | | 32.401 | 1.931 | | 16.780 | <0.001 |
| Gender | Male (ref. Female) | 1.559 | .399 | .053 | 3.905 | <0.001 |
| Year of study | | −.910 | .169 | −.073 | −5.376 | <0.001 |
| Affiliation | | −.436 | .251 | −.023 | −.174 | 0.082 |
| Membership in club activities or circles | | .151 | .274 | .008 | .553 | 0.581 |
| Satisfaction with diet | | 1.582 | .799 | .028 | 1.981 | 0.048 |
| Meals with a focus on nutritional balance | | 2.167 | .405 | .076 | 5.353 | <0.001 |
| Replacing meals with snacks | Yes (ref. No) | 4.948 | .474 | .145 | 10.435 | <0.001 |
| Frequency of breakfast | | −.121 | .147 | −.012 | −.823 | 0.411 |
| Sleep quality | | 3.405 | .406 | .116 | 8.397 | <0.001 |
| Exercise frequency | | −.623 | .383 | −.097 | −6.858 | <0.001 |
| Health status over the past month | | −3.794 | .626 | −.085 | −6.060 | <0.001 |
| Satisfaction with college life | | 4.292 | .663 | .089 | 6.475 | <0.001 |

*B, unstandardized coefficient; SE, standard error; β, standardized coefficient. Dependent variable: IAT total score (continuous; range 20–100). The direction and significance of all key predictors were consistent with those obtained in the primary multinomial logistic regression analysis (Table 4), supporting the robustness of the categorization approach.*
