# Supplementary material for: Internet Addiction and Problem Gambling Among Japanese University Students: Comorbidity and Lifestyle Correlates
Source: Behav Sci (Basel). 2026 May 8;16(5):728. doi: 10.3390/bs16050728 (PMC13203873; doi:10.3390/bs16050728)
Supplement: Supplementary file 1 [file behavsci-16-00728-s001.zip › Supplementary Table S2.pdf]

Supplementary Table S2. Binary logistic regression analysis with SOGS dichotomized as 0 (no problem) vs. ≥1 (any gambling problem) as the dependent variable (sensitivity analysis).

Variable	Category	OR	95% CI		p-value
			Lower	Upper	
IAT score (continuous)		1.016	1.005	1.025	0.002
Year of study	(ref. 4th year)				0.128
	1st year	1.115	0.779	1.597	0.588
	2nd year	0.829	0.581	1.182	0.349
	3rd year	0.714	0.497	1.028	0.111
Satisfaction with diet	Satisfied (ref. not satisfied)	1.787	0.983	3.248	0.057
Meals with a focus on nutritional balance	Yes (ref. no)	1.261	0.940	1.691	0.125
Replacing meals with snacks	Yes (ref. no)	0.728	0.507	1.047	0.092
Parental gambling problems	(ref. both parents)				0.004
	No parental problems	2.032	1.142	3.613	0.017
	Either parent	4.818	1.358	17.087	0.015

OR, odds ratio; CI, confidence interval. IAT was entered as a continuous variable. The direction and significance of key predictors (IAT score and parental gambling history) were consistent with the primary multinomial logistic regression analysis (Table 5).
