# Supplementary material for: Internet Addiction and Problem Gambling Among Japanese University Students: Comorbidity and Lifestyle Correlates
Source: Behav Sci (Basel). 2026 May 8;16(5):728. doi: 10.3390/bs16050728 (PMC13203873; doi:10.3390/bs16050728)
Supplement: Supplementary file 1 [file behavsci-16-00728-s001.zip › Supplementary Table S3.pdf]

Supplementary Table S3. Binary logistic regression analysis with SOGS dichotomized as 0–4 (non/subclinical) vs. ≥5 (problem gambling) as the dependent variable (sensitivity analysis).

Variable	Category	OR	95% CI		p-value
			Lower	Upper	
IAT score (continuous)		1.026	1.011	1.041	<0.001
Year of study	(ref. 4th year)				0.882
	1st year	1.224	0.435	3.441	0.552
	2nd year	1.299	0.477	3.538	0.438
	3rd year	1.132	0.399	3.213	0.731
Satisfaction with diet	Satisfied (ref. not satisfied)	1.140	0.207	6.278	0.764
Meals with a focus on nutritional balance	Yes (ref. no)	0.848	0.369	1.948	0.508
Replacing meals with snacks	Yes (ref. no)	0.887	0.289	2.726	0.677
Parental gambling problems	(ref. both parents)				<0.001
	No parental problems	2.285	0.671	7.788	0.019
	Either parent	7.230	1.695	30.847	<0.001

OR, odds ratio; CI, confidence interval. IAT was entered as a continuous variable. Given the small sample size of the problem gambling group (n = 14), these results should be interpreted as exploratory and preliminary. The association between IAT score and problem gambling risk remained statistically significant, consistent with the primary analysis (Table 5).
